# Why Gerontology Needs Anthropology: Toward an Applied Anthropological Gerontology

**DOI:** 10.3390/socsci13010004

**Published:** 2023-12-20

**Authors:** Britteny M. Howell, M. Aaron Guest

**Affiliations:** 1 Division of Population Health Sciences, University of Alaska Anchorage, 3211 Providence Dr., Anchorage, AK 99508, USA; 2 National Resource Center for Alaska Native Elders, University of Alaska Anchorage, 2702 Gambell St., Anchorage, AK 99508, USA; 3 Center for Innovation in Healthy and Resilient Aging, Arizona State University, 500 N. 3rd St, Phoenix, AZ 85004, USA;

**Keywords:** applied anthropology, gerontology, aging, cultural factors, health care policy, health outcomes

## Abstract

In this essay, we argue that gerontologists should increase their engagement with anthropologists to increase transdisciplinary collaboration, fulfill the interdisciplinary promise of gerontology as a field, and to ensure the work of anthropologists is formed by, and employed in, situations where meaningful engagement with practitioners and policymakers can lead to social change. Anthropology is the study of human societies in historical, biological, and sociocultural context, comprising a holistic field of study that can contribute unique methods, approaches, and theories to the field of gerontology. Although increasing amounts of anthropological scholarship have focused on older adulthood, this critical work of anthropologists still needs to be utilized by those in positions of power to enact change. Furthermore, the work conducted by anthropologists of aging has not consistently been recognized as anthropological scholarship. Therefore, a notable gap exists between the promise of the anthropology of aging and the utilization of the field, its findings, and engagement with the broader gerontological academy. As such, the contributions of anthropology to aging scholarship and the resulting reduction in inequities in the aging experience are not always adequately recognized. By examining the history of anthropology’s engagement with aging and the lifecourse, we argue for a more applied anthropological gerontology. We conclude with a call to action to ensure that anthropological gerontology is seen as a fundamental branch of scholarship, both within anthropology and gerontology, which can be used to improve the lived experiences of older adults globally.

## 1. Introduction

Each nation is experiencing growth in the size and proportion of the older adult population. In 2020, the number of people aged 60 years and older outnumbered children under the age of 5 for the first time in history (World Health Organization 2022). In the United States (U.S.), nearly 17% of the population is aged 65 or over and this proportion is growing (Caplan 2023). It is well known among gerontologists and anthropologists that there is significant variation in the aging experiences of people worldwide. Variations in age-related changes are so heavily influenced by environmental, genetic, sociocultural, and political–economic factors that older adulthood is the most heterogeneous phase of the lifespan, resulting in significant health disparities (Dannefer 2003). The increasing population of older adults around the world requires transdisciplinary efforts to ensure culturally appropriate interventions to mitigate such disparities, resist ageism, and provide care that recognizes the needs and wishes of older persons. Since the age-related changes occurring throughout life vary, anthropologists have a crucial role to play in documenting these social and cultural shifts. Sometimes their efforts are successful, resulting in research that influences policy and other societal changes that can improve the lives of older adults.

However, far too often, the vital work of anthropologists lacks utilization by those in positions of power who can enact change (Rylko-Bauer et al. 2006). Christine Fry suggests this discrepancy might be because anthropological work has long been relegated to the “niche” of documenting cross-cultural variation (Woodward and Culbert 2019), rather than as a possible avenue for providing important data and insights that might inform the practice of gerontology. Whether it is due to the inaccessibility of the work, a perceived lack of generalizability, a lack of dissemination, unfamiliarity in applicable fields, or omission, the work of anthropologist is often underutilized in improving the lives of older persons. Here we suggest that an *applied anthropological gerontology* may address these concerns, where meaningful engagement between anthropologists, gerontologists, public health practitioners, policymakers, and others may bridge the gap between anthropological research and social change.

In this paper, we use the term *anthropology* in the British/European tradition to refer specifically to *sociocultural anthropology* rather than the American usage of the term, which tends to take a multi-field approach, including biological, cultural, archaeological, and linguistic anthropology (Layton and Kaul 2006). We also use the term *applied anthropology* in this paper to refer to the pragmatic engagement of anthropological knowledge and skills to address contemporary problems (Rylko-Bauer et al. 2006). Lastly, we use the term *transdisciplinary* in this paper because it prioritizes thinking about problems as whole systems rather than examining issues within the confines of disciplinary boundaries. This term better describes our expectations of the field of gerontology than the term interdisciplinary, which attempts to link two or more disciplines into a coherent whole. Instead, transdisciplinarity does more than merely draw disciplines together, as it requires a common perspective and way of thinking that “transcends” disciplinary frameworks (Choi and Pak 2006). In this paper, we argue for an applied anthropological gerontology by first providing a brief history of these two fields, including some examples where the anthropological scholarship of aging has been successfully applied into gerontological practice. We conclude by providing a call to action for increased engagement between gerontologists and anthropologists, along with its documentation in the academic literature, to assure all relevant scholarship is applied to improve older people’s lives.

### 1.1. Defining Gerontology

The term *gerontology* was coined in 1905 as the scientific study of old age and was increasingly used as an interdisciplinary framework for scientific inquiry as early as the 1930s (Achenbaum and Levin 1989). The Gerontological Society (GSA) was developed in 1945 and began publishing academic journal articles in this burgeoning field in 1946. Although gerontology currently describes itself as transcending disciplinary boundaries (Ferraro 2018), there has been a long history of disciplinary silos within the field. In the 1940s, experts in aging began dividing the field into as few as three and as many as nine different subfields, often separating the biological, social, and psychological aspects of aging.

Despite such specializations within gerontology, there are several common threads within the field, especially social gerontology. According to Ferraro (2013), these include viewing aging through a lifecourse perspective as a heterogeneous life process, as a multi-faceted, multidimensional source of change over time that is influenced by genetics and cumulative dis/advantage. A lifecourse perspective on aging encourages gerontologists to view aging outcomes as partially (sometimes strongly) determined by the accumulation of advantage or disadvantage, those beneficial or adverse experiences accrued in early life, compounded by societal views toward aging, which are often ageist. Ageism refers to the stereotypes, prejudice, and discrimination towards others, or oneself, based on age (World Health Organization 2021). Combating ageism has become a major thread throughout gerontology, providing a fruitful avenue of inquiry for breaking down gerontological silos and opening up a space for an applied anthropological gerontology.

For example, current transdisciplinary health-focused efforts in gerontology such as the *Reframe Aging Initiative*, housed within the GSA, are working to change ageist perceptions of older adults by modifying how we describe and discuss aging individuals and their effects on society (Guest and Peckham 2022; Sweetland et al. 2017). These efforts were developed through a multi-year national framework analysis that included substantial input from anthropologists and aimed to discover how Americans discussed aging, how older adults discussed themselves, and to provide a long-term social change to increase the understanding of aging, which involved significant contributions from anthropologists. The results are a series of best practices and recommendations including (1) avoiding terminology that ‘others’ the population or invoking stereotypes, instead using neutral (e.g., older persons) and inclusive language, (2) focusing on affirmative traits of older persons, not solely negative, (3) avoiding words that indicate intergenerational discord, (4) avoiding generic descriptors and appeals, and (5) focusing on affirmative aspects of changing populations (i.e., avoid catastrophic terms). These and other current efforts by gerontologists to combat ageism and other negative factors that contribute to the treatment of older adults in society provide potential avenues for an applied anthropological gerontology.

### 1.2. Defining the Anthropology of Aging

The anthropological study of aging, or the *anthropology of aging*, has a rich and complex history, shaped by various theoretical and methodological approaches, although it developed more than 30 years after the field of gerontology. Several important, comprehensive records have been written about the anthropology of aging (see, for example, Cohen 1994; Fry 1981; Nydegger 1981; Perkinson and Solimeo 2014; Sokolovsky 2020); here, we provide a brief overview of the history of the development of an anthropology of aging, highlighting some of the critical theoretical and methodological contributions to its application.

Although anthropological interest in aging can be traced to the early twentieth century, with the work of scholars such as Franz Boas and Alfred Kroeber, one of the earliest anthropologists to focus specifically on aging was Ruth Benedict. Benedict argued that the emphasis on interdependence and family relationships in Japan shaped the experience of aging, with older adults relying on family members for support and care (Benedict 1934). In the mid-twentieth century, anthropologists began conducting cultural aging studies, comparing aging experiences across different societies. Emerging in the 1960s alongside the burgeoning field of social gerontology (see Marshall and Clarke 2007), the anthropology of aging finally became a subfield in its own right.

The late arrival of a focus on aging within the field of anthropology perplexed Margaret Clark, who noted that anthropologists relied heavily on the stories and experiences of older adults “as informants for a good deal of their cultural data” and because “anthropologists have long claimed the study of cultural patterning of the human life cycle,” including early-life transitions and various rites of passage (Clark 1967, p. 55). Whereas the aforementioned fields of biology and psychology quickly adapted to exploring the experience of aging, and the field of social gerontology would arise from sociology, anthropology would not begin its focus on aging in earnest for several more decades. Clark upbraided anthropologists for neglecting the life stages between achieving adulthood and death, except for a few ethnographies from the age-graded societies and so-called “gerontocracies” of Australia and Africa. She suggests there were two main reasons that anthropologists ignored this stage of life: (1) prevalent American (and Western) negative attitudes toward aging and (2) the concept of personality as immutable. Since anthropologists are products of their own cultures, they may have been subconsciously selected for personal, general, and public interest. The field of psychology may also have influenced anthropologists during this time to believe that personality is fixed in adulthood, a concept generally referred to as *continuity theory* by gerontologists (Atchley 1989) and *permanent personhood* by anthropologists (Lamb 2014). Understanding the history of engagement of anthropology and older adulthood, particularly as it relates to an area of inquiry, is necessary to fully grasp the division that exists to this day.

## 2. Applied Anthropology of Aging

### 2.1. Some Early Examples

Several researchers, including Christie Kiefer and Michael Angrosino, addressed Clark’s call for anthropology that engages more meaningfully with aging. Kiefer’s (1971) work sought to inform the nascent field of gerontology about the specific training and experiences that anthropologists can bring to the study of aging, as well as convince anthropologists that studying this stage of life is worthwhile and rewarding. She argued for the “triangulation” of aging, viewing the concept from the viewpoint of several disciplines, such as anthropology, which provides a methodology for studying ethnicity as a process, and gerontology, which takes a developmental perspective of life changes. Likewise, Angrosino (1976) argued that the methods and perspectives of anthropology could meaningfully contribute to the field of gerontology and cross-cultural comparative studies.

Such early persuasive works calling for an anthropology of aging led to more ethnographies of older adults and articles theorizing about personality and other changes throughout the lifecourse. Still, collaborative work with gerontologists largely lacked in the literature for several more decades. In the latter half of the twentieth century, anthropologists began to adopt a lifecourse perspective, exploring how known historical and social context shape aging. However, few of these contributions constitute *applied* anthropologies of aging. Cowgill and Holmes’ (1973) *Aging and Modernization* provided a collection of chapters intending to utilize anthropological knowledge and engage with the burgeoning field of gerontology. Still, the seven sociologists, eight anthropologists, two psychologists, and one social worker who contributed to the volume all wrote separate chapters without apparent transdisciplinary collaboration in their work or demonstration of application to the field.^1^

Clark and Anderson’s (1967) seminal work, *Culture and Aging*, begins with a preface indicating that this project was a radical departure from the “customary procedures” of anthropology, where anthropologists “rarely relied upon other specialists in the course of their work” (p. vii). This work is foundational to our field, as Clark and Anderson provide an early example of how we can contribute to a multidisciplinary team approach where anthropologists “apply the concepts they have developed to the study of complex urban societies” (p. viii) in collaboration with geriatricians and gerontologists for a broad readership including not just anthropologists but also other social scientists, social workers, senior service professionals, legislators, and clinicians.

Another notable example is Gwendolyn Safier’s (1975) work training nursing students in a geriatrics course on anthropological and sociological principles to improve their rapport with older patients. In collaborating with gerontologists and geriatric healthcare professionals, Safier aimed to ensure nursing students learned to recognize the influence of their own culture on their interactions with patients, the role of their patient’s culture, and the interrelationships between older adults and their communities. However, as Safier presents, case studies demonstrating an applied anthropology of aging are challenging to locate in the anthropological literature before the 1990s.

In 1978, the international non-profit Association for Anthropology, Gerontology, and the Lifecourse (AAGE) was established “as a multidisciplinary group dedicated to the exploration and understanding of aging within and across the diversity of human cultures” (AAGE 2023) and began publishing the open-access *Anthropology & Aging* journal. Around this time, the Society for Applied Anthropology began publishing its practice-focused journal for anthropologists working outside academia, *Practicing Anthropology*. The following year, Myerhoff and Simić’s (1979) *Life’s Career—Aging: Cultural Variations on Growing Old* was published, a volume that brought together the work of five anthropologists through the Andrus Gerontology Center at the University of Southern California. This work appears to be unique at that time not only for its collaborative efforts between anthropology and gerontology but also because each of the contributing authors had conducted fieldwork among peoples who constituted their own ethnic group, providing early examples of *insider ethnography* that was reflexive about the role of the investigator’s social position and identity.

The 1980s brought more ethnographic research on older adults, including the edited works of Christine Fry (Fry 1980, 1981). In Fry’s *Dimensions: Aging, Culture, and Health* (Fry 1981), Jennie Keith and Corrine Nydegger provide chapters suggesting pathways for more closely integrating anthropology and gerontology. Keith (1981) offers ways for anthropologists to mine the published ethnographies of older adults and ensure data are being utilized to “suggest variables and hypotheses” to inform anthropological theory. Nydegger (1981) approaches the issue from the field of gerontology, where she identifies a problem that plagues our field to this day: anthropology tends to be more theory-focused, while gerontology may be more problem-focused. Nydegger points out that this problem provides an exciting opportunity for traditionally underfunded disciplines, like anthropology, to contribute to transdisciplinary applied work.

### 2.2. Recent Exemplars

There are, of course, several more recent examples demonstrating the potential of a transdisciplinary approach to old age utilizing anthropology and gerontology. However, it was not until the 1990s, with the proliferation of medical anthropology as a sub-field, that we began to see substantially more applied work with older adults. Jay Sokolovsky’s (1990) edited volume and subsequent editions have contributed much to the discourse between anthropologists and gerontologists.

An important example of the successful merging of gerontology with applied anthropology is the work of Iris and Berman (1998). As part of a multidisciplinary team on the *Aging in Chicago Project*, these anthropologists describe their experiences translating their ethnographic work to other disciplines, integrating ethnographic findings with different data types, and applying them to shape policy. Another notable example is the work of Nancy Schoenberg, which focused on applying ethnographic knowledge among Black older adults to improve health outcomes related to diabetes (Schoenberg et al. 1998a, 1998b) and hypertension self-management (Schoenberg 1997, 1998). Schoenberg also engaged in work focused on cancer and chronic illness among rural older adults in the United States for over twenty years (Schoenberg et al. 2014). Her work has examined culture’s role in healthcare decision making and the development of interventions to improve health. Her work provides a blueprint for how anthropologists can engage with other professionals to adapt and implement health promotion interventions using an ethnographic lens and principles of implementation science (Schoenberg and Snell-Rood 2019).

Likewise, the work of Jean Schensul has long guided how to engage in applied ethnographic methods to improve the quality of life for older adults (Schensul 2023; Schensul et al. 2006). Others, such as Peggy Perkinson (2008), have focused on the cultural dimensions of aging to develop tailored interventions. She has used her anthropological background to develop community-based collaborations to improve the health of older persons (Perkinson 2012). Scholars such as Freidus and Shenk (2020); Freidus et al. (2021) have also recently used anthropological approaches to document and advance COVID-19 prevention strategies. In these instances, anthropologists have sought to inform the care of older persons by applying anthropological methods with specific health outcomes in mind. These works address the needs of aging populations by engaging with experts across various disciplines to ensure equitable care.

It is clear that anthropologists have contributed much to the ethnographic literature on the experience of aging and the treatment of older adults around the globe. As the above examples show, anthropological perspectives can also be leveraged in gerontology to improve robust integration of culture into aging research. However, aside from these and other notable examples such as the work of Sharon Kaufman (Kaufman 1986, 1995, 1997), Robert Rubenstein (see Clark-Shirley et al. 2020; Rubinstein 1992, 2012), Janelle Taylor (O’Hare et al. 2022; Taylor et al. 2023; Vig et al. 2017), and others, it is less clear the degree to which anthropological contributions have been applied to influence gerontology, or adapted and applied by gerontologists meaningfully.

## 3. Striving towards an Applied Anthropological Gerontology

The intent of this article is not to exhaustively review or overly critique the fields of gerontology or anthropology, but to encourage us all to consider what an applied and engaged anthropological gerontology may entail. Case studies and other examples demonstrating an applied anthropological gerontology remain challenging to locate in the academic literature. An argument could be made that the fault lies with gerontologists for not picking up on the contributions of anthropologists for decades. Indeed, as a field that describes itself as interdisciplinary (K. F. Ferraro 2018), there must be some onus on gerontologists to ensure engagement with existing scholarship. But it may also be the case that ethnographies and critical articles using highly theoretical language make anthropology inaccessible to the uninitiated. A required disciplinary-understanding threshold may exist to adequately engage with the anthropological literature (Scheper-Hughes 2009), one that is not easily achieved and may sometimes prevent collaboration.

To overcome such hurdles may require anthropologists to engage more closely with the work of gerontologists, biomedical researchers, and geriatricians. Likewise, gerontologists would benefit greatly from increased collaboration with anthropologists and utilizing transdisciplinary frameworks. Cohen et al. (2019) points out that much biomedical and geriatric work frames the research questions of gerontologists around age-related *declines* and *deficits*. Biomedical practitioners also approach aging as a problem to be solved, often with the goal to prolong life, sometimes at all costs. However, as anthropologists and many social gerontologists know, the wishes of older adults are often centered around different goals, such as maintaining autonomy, social relationships, generativity, and avoiding suffering (Howell et al. 2021; Van Leeuwen et al. 2019). This provides an excellent avenue for anthropologists to help expand how gerontologists view and utilize the lifecourse perspective. When anthropologists “tack” across disciplines (Park and Littleton 2013), such as geriatric medicine or public policy, the field will continue to see progress toward addressing issues of anthropological relevance and ageism in healthcare.

The anthropology of aging aims to deepen our understanding of how humans age through place and time. Therefore, anthropologists contribute to public health research on current demographic and economic trends by studying the changes in fertility, life expectancy, and population-age structures (see Bachrach 2013). However, few anthropologists consider themselves gerontological anthropologists or anthropologists of aging. Given the breadth of theoretical and methodological expertise anthropologists represent, a concerted effort to engage in aging scholarship with gerontologists could result in a significant reduction in inequities. For example, engagement with the participant-forward- and participant-centered approaches of anthropology, through ethnography and participant observation, could open the door to new avenues of research and discovery. Anthropologists can improve the design of health interventions by ensuring they are adaptable, interactive, and support health justice by drawing attention to forms of expertise, knowledge, and action grounded in communities (Yates-Doerr et al. 2023). Likewise, understanding the needs of older individuals through their eyes—often an aim, but rarely successfully achieved in gerontology—would assist researchers in identifying the actual barriers to optimal health, rather than just what the researchers perceive. Indeed, innumerable potential contributions exist from an applied anthropology of aging, particularly as we focus on the implications of the most significant proportion of older adults that the world has ever known.

For example, anthropology has a well-developed scholarship of various systems of power and hierarchy that affect macro- and micro-environments, including the healthcare system, public policy, sociocultural beliefs, and individual lived experiences. An applied anthropological gerontology could take a transdisciplinary approach to further elucidate the ways that systems of power influence communities of older adults and vice versa, such as examples of grassroots efforts by older adults to influence public policy and combat ageism.

Collaborative efforts are needed now more than ever; the public health and public policy literature is riddled with language that suggests this “booming” population of seniors is a burdensome “silver tsunami” hoarding resources and damaging local economies (Gendron et al. 2016). Demographers and epidemiologists have been forecasting changes in our aging society for years. Still, public policy has largely ignored this growing segment of our population, which could be further remedied by continued engagement with anthropologists (Sabloff 2011).

## 4. Conclusions and Future Directions

Our intention has not been to insinuate that there is no anthropology of aging scholarship nor that applied, scholarly work that blends gerontological and anthropological approaches does not exist. Rather, we have aimed to show the continued need for this scholarship and that it holds great potential in advancing our work toward an equitable society for all ages. Similarly, we have not purposefully excluded any work that may fulfill this call. In collecting our thoughts to develop this piece, we found ourselves looking toward the literature and disheartened that such applied gerontological work is either excluded from the anthropological scholarly literature, and thus less likely to be accessed by anthropologists, or not labeled as such (i.e., keyword searchable). There is a need to encourage the submission of such work to anthropological and gerontological journals and remove the barriers that may prevent it from being marked or accepted as anthropological scholarship. Likewise, as a discipline which has made our distinguishing feature the interdisciplinarity of our scholarship, gerontology has greater opportunity to engage with the existing anthropology of aging literature.

Because aging occurs collectively and individually, an applied anthropological gerontology interrogates the political, environmental, social, and economic conditions contributing to the health and other disparities for older adults worldwide (Lynch and Danely 2013). Much like gerontology, an applied anthropological gerontology would represent a transdisciplinary knowledge enterprise that engages across disciplinary boundaries and contributes much to our understanding and approach to aging. By applying anthropological insights to an aging world, we can reveal the cultural values, norms, and expectations that shape people’s lifecourse experiences. As exemplars in this field have shown (i.e., Schoenberg, Taylor, Perkinson, Schensul, and others), engaging in the cultural aspect of health can result in more positive health outcomes. Additionally, ethnographic work can help provide examples of the essential contributions to society that older adults offer and help change the public discourse from a deficit-based to an asset-based perspective of older adults and their contributions to society. Since many questions about human variation can be addressed in older adulthood, there is a necessity to see more anthropologists contributing to this vital work and encouraging students to consider careers in gerontology, geriatrics, or an applied anthropology of aging (see Harman 2005). Collectively, greater cohesion and collaboration among gerontology and anthropology would result in more cohesive, and applicable, understandings of health and wellbeing in older adulthood.

The anthropology of aging and an applied anthropological gerontology should be fundamental branches of scholarship used to improve the lived experiences of older adults globally. Accomplishing this aim requires a concerted effort on the part of anthropologists and gerontologists. There must be efforts to bridge the disciplinary silos by engaging in purposeful transdisciplinary conversations and collaborations. Such efforts may include specific conferences and meetings that actively recruit anthropologists, gerontologists, and/or geriatricians to attend and exchange dialogue. Doing so will require a level of base-setting and the willingness to abandon disciplinary-specific terminology in the interest of communication. From these and other efforts, future opportunities involving the co-production of knowledge and intervention to improve health through a cultural-based understanding can emerge. Through work to remove barriers to publication, it can then be shared and accessed by scholars across fields. Ultimately, advancing this work will require the development of degree programs and pedagogical strategies that seek to integrate anthropological and gerontological methods and theories. The development of innovative, cross-disciplinary coursework has the potential to interest an entire generation of emerging scholars in the work of applied anthropology of aging. If we can achieve these aims, the result will be a more equitable world for all ages.

## Data Availability

No new data were created or analyzed in this study. Data sharing is not applicable to this article.

## Footnotes

1 Interestingly, this book was marketed as part of a “sociology series” by the publisher, rather than as anthropology.
